# A Monovalent and Trivalent MVA-Based Vaccine Completely Protects Mice Against Lethal Venezuelan, Western, and Eastern Equine Encephalitis Virus Aerosol Challenge

**DOI:** 10.3389/fimmu.2020.598847

**Published:** 2021-01-19

**Authors:** Lisa Henning, Kathrin Endt, Robin Steigerwald, Michael Anderson, Ariane Volkmann

**Affiliations:** ^1^Battelle Memorial Institute, Columbus, OH, United States; ^2^Bavarian Nordic GmbH, Martinsried, Germany

**Keywords:** modified vaccinia Ankara (MVA), vaccines, efficacy, alphavirus, equine encephalitis

## Abstract

Venezuelan, eastern and western equine encephalitis viruses (EEV) can cause severe disease of the central nervous system in humans, potentially leading to permanent damage or death. Yet, no licensed vaccine for human use is available to protect against these mosquito-borne pathogens, which can be aerosolized and therefore pose a bioterror threat in addition to the risk of natural outbreaks. Using the mouse aerosol challenge model, we evaluated the immunogenicity and efficacy of EEV vaccines that are based on the modified vaccinia Ankara-Bavarian Nordic (MVA-BN^®^) vaccine platform: three monovalent vaccines expressing the envelope polyproteins E3-E2-6K-E1 of the respective EEV virus, a mixture of these three monovalent EEV vaccines (Triple-Mix) as a first approach to generate a multivalent vaccine, and a true multivalent alphavirus vaccine (MVA-WEV, Trivalent) encoding the polyproteins of all three EEVs in a single non-replicating MVA viral vector. BALB/c mice were vaccinated twice in a four-week interval and samples were assessed for humoral and cellular immunogenicity. Two weeks after the second immunization, animals were exposed to aerosolized EEV. The majority of vaccinated animals exhibited VEEV, WEEV, and EEEV neutralizing antibodies two weeks post-second administration, whereby the average VEEV neutralizing antibodies induced by the monovalent and Trivalent vaccine were significantly higher compared to the Triple-Mix vaccine. The same statistical difference was observed for VEEV E1 specific T cell responses. However, all vaccinated mice developed comparable interferon gamma T cell responses to the VEEV E2 peptide pools. Complete protective efficacy as evaluated by the prevention of mortality and morbidity, lack of clinical signs and viremia, was demonstrated for the respective monovalent MVA-EEV vaccines, the Triple-Mix and the Trivalent single vector vaccine not only in the homologous VEEV Trinidad Donkey challenge model, but also against heterologous VEEV INH-9813, WEEV Fleming, and EEEV V105-00210 inhalational exposures. These EEV vaccines, based on the safe MVA vector platform, therefore represent promising human vaccine candidates. The trivalent MVA-WEV construct, which encodes antigens of all three EEVs in a single vector and can potentially protect against all three encephalitic viruses, is currently being evaluated in a human Phase 1 trial.

## Introduction

The alphavirus genus of viruses comprises arthropod-transmitted, enveloped positive-sense, single-stranded RNA viruses that includes a diverse group of at least 30 species (1). Venezuelan equine encephalitis virus (VEEV), eastern equine encephalitis virus (EEEV), and western equine encephalitis virus (WEEV) comprise the encephalitic group that causes overt disease of the central nervous system in both equids and humans. Human infection typically results in an incapacitating disease characterized by fever, headache, lymphopenia, myalgia, and malaise (2–5). Additionally, severe neurological disease, which includes fatal encephalitis, can occur with a fatality rate estimated to be ≤1% for VEEV, 8–15% for WEEV, and 30–70% for EEEV (4, 5). There is concern for their use as bioweapons because of the ease of production, high infectivity, potential for aerosolization, and capacity to induce acute, febrile, incapacitating disease. The United States (US) government considers some strains of VEEV and EEEV as potential bioterror agents, which are regulated by the Centers for Disease Control Select Agent Program and the US Department of Agriculture. Moreover, outbreaks of emerging and re-emerging arboviruses, such as dengue, West Nile, chikungunya, Zika and also equine encephalitis appear to become more common, such as seen with the unusually high number of EEEV cases in the US in 2019 (6).

There is currently no licensed vaccine for human use for encephalitic alphaviruses available. Live-attenuated and formalin-inactivated vaccines utilized as Investigational New Drugs (IND) have limitations in effectiveness or undesirable reactogenicity (7–10). For example, live-attenuated VEEV (TC-83), though inducing neutralizing antibodies in about 80% of humans after a single vaccination, induces moderate flu-like symptoms in 40% of vaccinees (11, 12), while inactivated EEV vaccines are poorly immunogenic, require three doses for immunization and annual boosters, and are not completely protective against aerosol challenge.

Modified Vaccinia Ankara (MVA) is a highly attenuated Vaccinia virus that is adapted to chicken embryo fibroblasts (13, 14). MVA-BN is further attenuated, replication deficient in humans and other mammals, including immunocompromised mice and non-human primates (NHPs) (15, 16), and is approved as safer smallpox vaccine in USA (JYNNEOS™), Canada (IMVAMUNE^®^) and the European Union (IMVANEX^®^). MVA-BN’s excellent safety profile in humans (17–22), the maintained capability to induce strong and durable cellular and humoral immune responses (23–27), and the capacity to express numerous foreign genes makes it an attractive vaccine platform. Indeed, a recombinant MVA-BN expressing filovirus antigens (Mvabea^®^) is approved in Europe as part of the two-dose regimen with a recombinant Adenovirus (Zabdeno^®^) for the prevention of Ebola virus disease.

Applying the MVA platform to the need for EEV vaccine development, monovalent MVA-BN based encephalitic alphavirus vaccine candidates MVA-VEEV, MVA-WEEV, and MVA-EEEV were generated that encode E3-E2-6k-E1 of VEEV (TrD strain), WEEV (71V-1658 strain), or EEEV (FL93-939NA strain), respectively, as well as a multivalent MVA-WEV (Trivalent) that encodes E3-E2-6k-E1 of all three alphaviruses (28). These vaccines were previously shown to afford protection against intranasal challenge of mice with homologous VEEV-TrD and heterologous WEEV-Fleming or EEEV-PE6 strains. While the data indicated that alphavirus neutralizing antibodies likely played a role in protection, some fully protected mice with low or no measurable neutralizing antibody titers suggested that additional immune parameters may be important (28).

In the studies summarized here, we have utilized aerosol challenge models in mice, since the inhalational route is the anticipated route of infection in an intentional release of the equine encephalitic viruses. Immunogenicity (neutralizing antibodies) and protective efficacy of monovalent MVA-VEEV, MVA-WEEV, MVA-EEEV, and trivalent MVA-WEV was evaluated against aerosol challenge with homologous VEEV-TrD, as well as heterologous VEEV-INH-9813, WEEV-Fleming, and EEEV-V105-00210 in BALB/c mice. As previous data indicated that neutralizing antibodies are not solely associated with protection against encephalitic alphavirus challenge (28–32), analysis of T cell responses was included in one of the studies discussed within this manuscript.

## Materials and Methods

### Animals

All procedures performed on animals including euthanasia criteria were approved by IACUC and Animal Care and Use Review Office (ACURO) and complied with all Ohio state and US federal guidelines. Female, 6–8 weeks old BALB/c mice (16–24 g) were procured from Charles River Laboratories (Raleigh, NC; Stone Ridge, NY). Mice were implanted with a programmable temperature transponder chip (IPTT-300, BMDS, Seaford, DE) injected subcutaneously prior to vaccination. Animals were anesthetized prior to injection of the temperature probe. All challenge and post-challenge activities were performed in the BSL-3 facility.

### Study Design

MVA-BN^®^ (ECACC cat no. V00083008) was used as backbone virus (15). Design and generation of vaccines were described previously (28). In Study 1, on Study Days 0 and 28, BALB/c mice were administered Tris buffered saline (TBS), MVA-VEEV, Triple-Mix (MVA-VEEV, MVA-EEEV, MVA-WEEV), or MVA-WEV (Trivalent) *via* the intramuscular (IM) route at a dose of 1x10^8^ TCID_50_ each, i.e. Triple-Mix contained 3x10^8^ TCID_50_ total vaccine. Animals were challenged at Study Day 42 with VEEV (TrD).

In Study 2, on Study Days 0 and 28, BALB/c mice were administered TBS, the respective monovalent vaccine (MVA-VEEV, MVA-WEEV or MVA-EEEV) or MVA-WEV (Trivalent) *via* the IM route at a dose of 1x10^8^ TCID_50_ and were challenged either with VEEV (INH-9813), WEEV (Fleming) or EEEV (V105-00210) on Study Day 42. In addition, animals in the VEEV (INH-9813) challenge group were vaccinated with the Triple-Mix (1 x 10^8^ TCID_50_ each construct).

### Inhalation Challenge

A nose-only exposure chamber (CH Technologies, Inc., Westwood, NJ) was utilized to administer a target aerosol dose of 50 PFU VEEV INH-9813, 250 PFU VEEV Trinidad Donkey (TrD), 11,284 PFU WEEV Fleming, or 15,394 PFU EEEV-V105-00210 to the mice. These target doses were chosen as they were highly lethal in control mice in natural history studies. The aerosol challenge was performed as previously described (33) with the exception that the nose-only exposure chamber was used that provides the ability to simultaneously challenge multiple mice with a homogeneous, small-particle aerosol. The Mass Median Aerodynamic Diameter (MMAD) of the aerosol was determined for at least one time point during each 10 min test with an Aerodynamic Particle Sizer^®^ Spectrometer (APS Model 3321 TSI, Inc., Shoreview, MN). The inhaled dose (PFU/animal) was determined using Guyton’s formula with the mean body weight of animals in each challenge run and concentration of virus in the nebulizer and impinger determined by the plaque assay. The average calculated inhaled doses were 34 PFU/animal (VEEV INH-9813), 162 PFU/animal (VEEV TrD), 9,309 PFU/animal (WEEV), and 15,493 PFU/animal (EEEV).

### Clinical Evaluation

Mice were monitored throughout the studies for clinical observations (e.g. morbidity, mortality, changes in hair coat, respiration, hunched posture). In addition, body weights and twice daily body temperatures (using temperature transponder as described above) were measured during the post-dosing and/or post-challenge periods.

### Neutralization Assay

Neutralizing antibody levels in serum were quantified using a plaque reduction neutralization test (PRNT). PRNT was conducted using VEEV TrD, WEEV Fleming, and EEEV-V105-00210 input virus on serum samples collected during the post-dosing period. Briefly, The PRNT utilizes serial dilutions starting at 1:10 (LOD for this assay is 10) of heat-inactivated (56°C for 30 min in a water bath) serum that is pre-incubated with a known amount of virus to allow neutralization to occur. The serum-virus dilutions were then plated onto a susceptible Vero E6 cell monolayer. Overlay medium was added to allow plaque formation prior to staining. The plaques were enumerated, and the titer was calculated as the reciprocal of the serum dilution neutralizing 50% of the input virus (PRNT_50_).

### Enzyme Linked ImmunoSpot *Assay*

T cell response in Study 1 was evaluated *via* the Enzyme Linked ImmunoSpot (ELISpot). Splenocytes were harvested from spleens collected from animals during the post-dosing period to evaluate the interferon gamma (IFNɣ) response to VEEV TrD E1 and E2 peptide pools (final concentration of 2 µg/ml per peptide in the assay well). Each primary peptide pool consisted of 20 15-mers overlapping by 9 amino acids. Five of the primary parent peptide pools were combined to prepare secondary parent peptide pools that were used for the ELISpot (Primary peptide pools obtained from Mimotopes Pty Ltd). Cells were stimulated with peptide pools and incubated on a plate that was coated with anti-IFNɣ antibody to capture secreted protein. Upon completion of the stimulation incubation, the cells were removed from the plate and a detection antibody was added to bind the plate-bound protein. The detection antibody was coupled to an enzyme that converts a substrate into a colored precipitate. After color development, the plates were scanned and counted using an automated plate reader.

### Viremia Assessment

Following challenge, viremia was measured by the plaque assay on Vero E6 monolayers. Whole blood was collected and centrifuged to obtain serum. Serum samples were added to culture plates. After incubation, overlay medium was then added to each well. After incubation, wells were stained with crystal violet and subsequently plaques were counted.

### Statistical Analysis

The proportion of surviving animals 21 days post-challenge was calculated for each group and Clopper-Pearson 95% confidence intervals were calculated. Pairwise two-sided Fisher’s exact tests were performed to determine whether the proportion of surviving animals was significantly different between each vaccination group and the appropriate control group. Kaplan-Meier estimates were calculated and plotted to estimate whether time to death was significantly different across all pairs of challenged groups.

Geometric means and 95% confidence intervals were calculated for PRNT titers and body temperature by group and time point. Assay measurements were log-transformed, and assay values reported as less than the limit of detection (LOD) were imputed as one-half the LOD (PRNT LOD = 10). The LOD for the plaque assay is as follows: 177 PFU/ml (VEEV INH-9813), 96 PFU/ml (EEEV), 259 PFU/ml (WEEV), and 213 PFU/ml (VEEV TrD). Analysis of variance (ANOVA) models were used to determine if there were statistically significant differences in PRNT titers or body temperature among the groups at each time point. Tukey’s multiple comparisons was performed to determine which groups were significantly different. For body temperature analysis, each animal served as its own control and change from post-vaccination baseline was compared at each post-challenge study day to determine significant differences among the groups.

For each of the VEEV peptide pools (E1 and E2), a two-sample t-test was performed to determine if the difference in mean response (SFU/million cells) between each possible pair of treatment groups was statistically significant on each of Days 28 and 42. In addition, t-tests were performed to determine if the difference in mean response for a vaccine statistically differed between Day 28 and Day 42 for each peptide pool. The Type I error for each test was controlled at no more than 5%.

## Results

### Homologous Protection After Venezuelan Equine Encephalitis Virus (Trinidad Donkey) Aerosol Challenge

**Figure 1:**
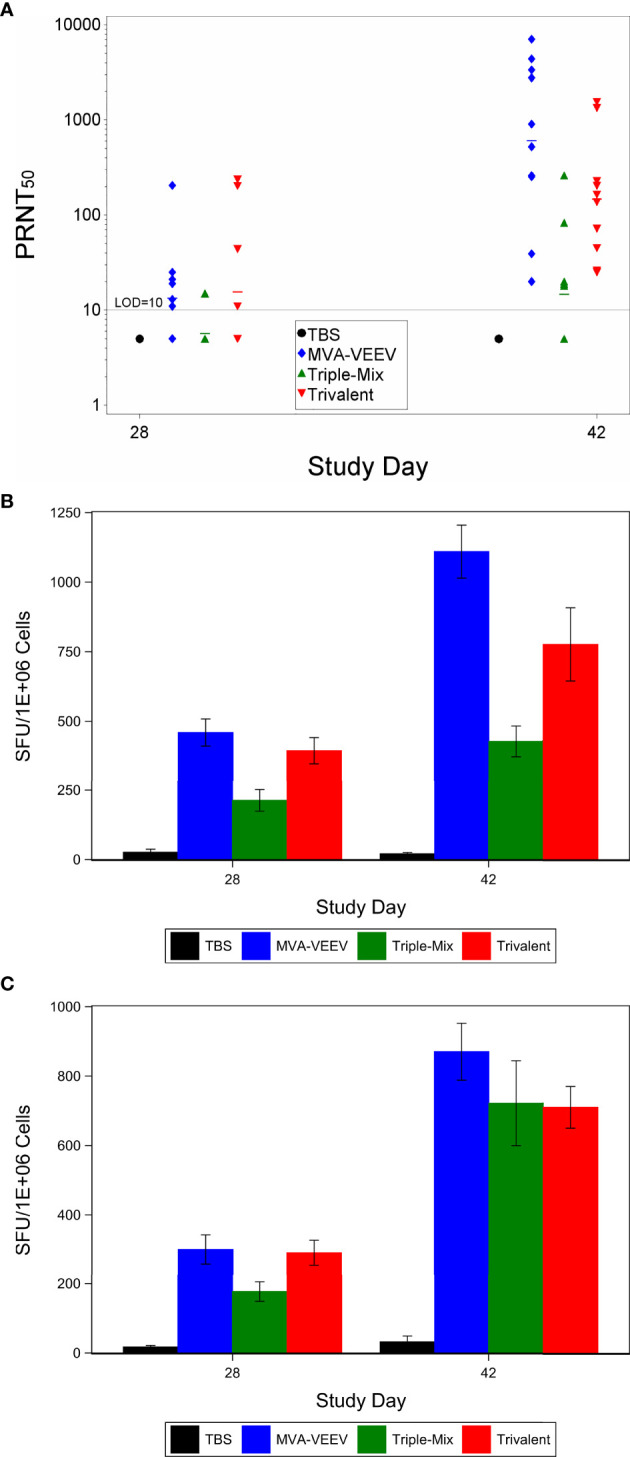
Immune Response After Immunization with MVA-VEEV, Triple Mix, or Trivalent Vaccines. Mice were immunized (IM) twice (Day 0 and 28) with the respective vaccine or TBS. PRNT_50_ **(A)** and Interferon gamma response (ELISpot) to the E1 peptide pool **(B)** and E2 peptide pool **(C)**. The second vaccination on Day 28 (Day 42) resulted in a significant increase in the neutralizing antibody titers compared to Day 28 (p < 0.05) in all three vaccination groups. In addition, the monovalent MVA-VEEV and Trivalent IFNγ response was significantly greater (p < 0.05) than the IFNγ response measured at Day 28 (E1 and E2 peptide pool) and Day 42 for the Triple-Mix group (E1 peptide pool). Ten animals per group were evaluated at each time point. **(A)** horizontal lines represent group mean values. Standard error of the mean is included in **(B, C)**.

The first study investigated the immunogenicity and efficacy of MVA based vaccine candidates against the homologous VEEV Trinidad Donkey strain encoded in monovalent MVA-VEEV as well as in Trivalent MVA-WEV. In addition, a mixture of all three monovalent EEV vaccines (Triple-Mix) was tested. This way, possible interference as previously suggested for EEV vaccines (34–36) could be evaluated and compared in a situation when the antigens are expressed by individual vaccines (Triple-Mix) or by the single MVA vector (MVA-WEV, Trivalent). Female BALB/c mice were vaccinated twice with the respective vaccine candidate at a four-week interval. Analysis of the neutralizing antibody response showed that a PRNT_50_ was observed for 6 of 10, 1 of 9 and 5 of 10 animals in the MVA-VEEV, Triple-Mix, and Trivalent groups, respectively, already four weeks after the first vaccination (Study Day 28, **Figure 1A**). Two weeks after second vaccination (Study Day 42) complete seroconversion was observed for all animals in the MVA-VEEV and the Trivalent groups and the majority (5 of 9) of the Triple-Mix vaccinated animals exhibited a PRNT_50_ titer, although there was a wide range in the magnitude of the responses with titers ranging from 18 to 1,550 (**Figure 1A**). The second vaccination resulted in a significant increase in the neutralizing antibody titers compared to Day 28 (p < 0.05) in all three vaccination groups. Overall, the peak PRNT_50_ of the monovalent MVA-VEEV [geometric mean titer (GMT) of 609] and the Trivalent (GMT 146) groups were significantly greater than the neutralizing antibody response measured for the Triple-Mix group (GMT 14.7), and no PRNT_50_ titer in control (TBS) animals was detectable at the time points assessed.

To investigate other arms of the immune system and considering recent studies that indicated a potential role of T cells in the protection against VEEV (37–40), cell mediated immune responses directed against VEEV (TrD) E1 and E2 were evaluated on Study Day 28 and 42 (**Figures 1B, C**). An IFNɣ response to VEEV (TrD) E1 and E2 peptide pools was observed for all mice of all three vaccine candidate groups already four weeks after the first vaccination (Study Day 28), with a significantly (p < 0.05) increased response two weeks after the second vaccination (Study Day 42). The MVA-VEEV group (460 & 1,110 mean SFU/10^6^ splenocytes) and Trivalent group (394 & 775 mean SFU/10^6^ splenocytes) exhibited significantly (p < 0.05) greater E1 peptide pool IFNɣ responses on Study Days 28 and 42 compared to the Triple-Mix group (213 & 428 mean SFU/10^6^ splenocytes), while with 870, 710, and 722 mean SFU/10^6^ splenocytes in the monovalent, Trivalent and Triple-Mix group, respectively, similar E2 peptide pool IFNɣ T cell responses were detected for all vaccinated groups on Study Day 42. Thereby, the potential immune interference seen in terms of neutralizing antibodies inferred by the Triple-Mix held true for E1, but not for E2 specific T cell responses.

**Figure 2:**
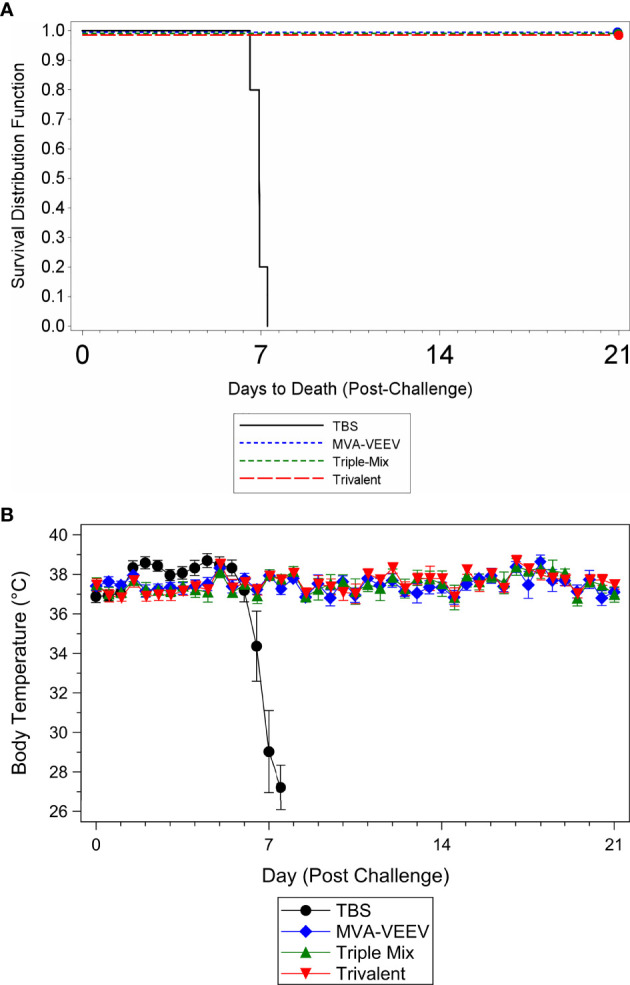
Survival and Body Temperature Changes after VEEV TrD Aerosol Challenge of Immunized Mice. Mice were immunized (IM) twice (Day 0 and 28) with the respective vaccine or TBS and then exposed to an aerosolized dose of VEEV TrD on Day 42. Kaplan-Meier plots of survival results **(A)** and changes in body temperature during the post-challenge period **(B)**. There was a significant difference in survival between the TBS group and all three vaccination groups (p < 0.01). In addition, there was a significant (p < 0.05) temperature decrease for the TBS control group **(B)** prior to succumbing to infection or meeting euthanasia criteria and a significant difference between the TBS control group and the vaccinated groups on Days 44–49 (p < 0.05). Ten animals per group were evaluated for survival and body temperature changes.

To determine whether vaccination with the different vaccine candidates protected against a homologous VEEV aerosol infection, mice were challenged two weeks after last vaccination (Study Day 42) with a lethal dose of VEEV (TrD), the homologous challenge strain identical to the structural proteins encoded by the different vaccine constructs. All animals in the MVA-VEEV, Trivalent and the Triple-Mix group survived the VEEV (TrD) challenge (**Figure 2A**) and were observed as normal with no changes in body weights (**Supplementary Figure 1A**) and body temperature (**Figure 2B**) throughout the post-challenge period. In contrast, all TBS control animals were observed with clinical signs including lacrimation, ruffled hair coat and hunched posture, showed a decrease in body weight (**Supplementary Figure 1A**), developed an elevated temperature 1–5 days post challenge followed by a significant (p < 0.05) temperature decrease (**Figure 2B**) and did not survive the viral infection. Consequently, statistically significant differences in survival was shown between the TBS group and all three vaccination groups (p < 0.01), while there were no statistically significant differences in time to death among any of the vaccination groups since all vaccinated animals survived. In addition, there was a significant difference in group mean body temperature between the TBS control group and the vaccinated groups on Days 44–49 (p < 0.05).

While several of the TBS control animals were viremic four days post challenge (Day 46; viral load range 1.52x10^5^ to 1.46x10^7^ pfu/ml), and in some instances at the terminal time point (2.33x10^2^ to 2.15x10^3^ pfu/ml), viremia could not be detected in any of the vaccinated animals at any time point during the post-challenge period. These results, in combination with survival and other parameters assessed (observations, body weights, body temperatures) confirmed that MVA-VEEV alone, in combination with two other MVA-based EEV vaccines, and also the Trivalent vaccine conferred complete protection against homologous VEEV (TrD) aerosol infection.

### Heterologous Protection After Venezuelan Equine Encephalitis Virus (INH-9813), Western Equine Encephalitis Virus (Fleming), and Eastern Equine Encephalitis Virus (V105-00210) Aerosol Challenge

**Figure 3:**
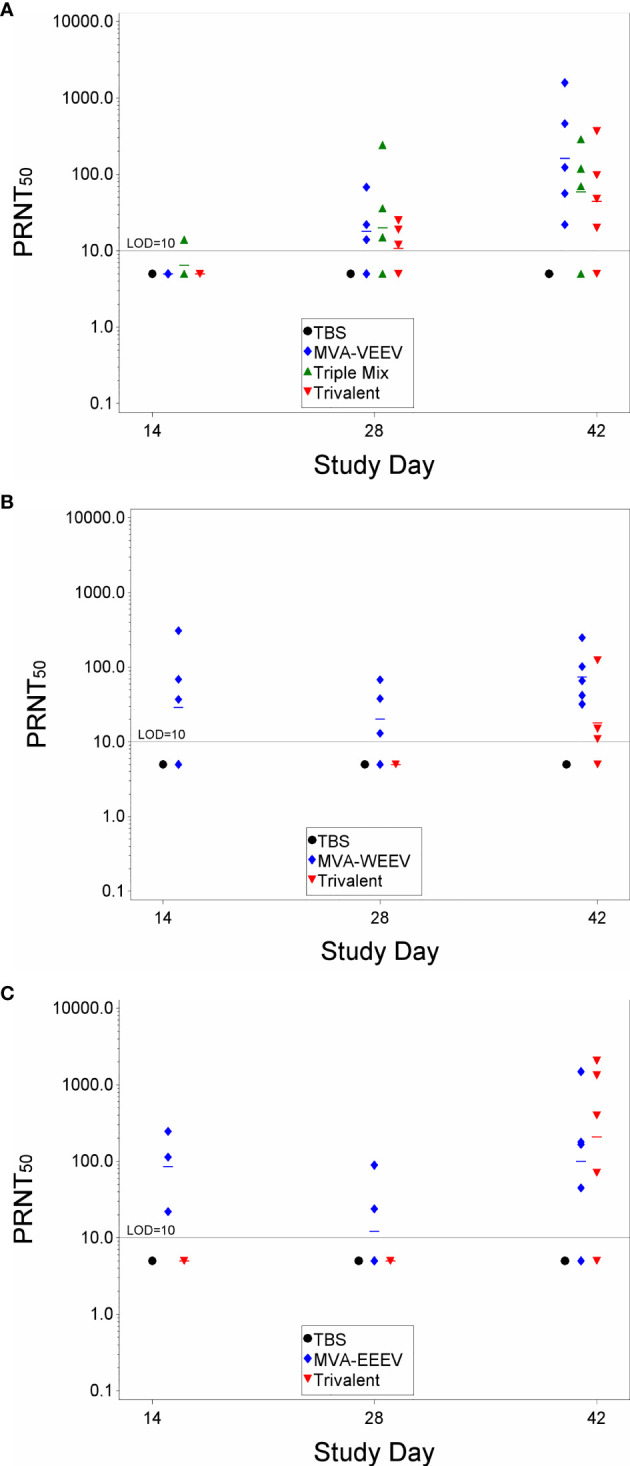
Immune Response After Immunization with Monovalent, Triple Mix, or Trivalent Vaccines. Mice were immunized (IM) twice (Day 0 and 28) with the respective vaccine or TBS. VEEV PRNT_50_ **(A)**, WEEV PRNT_50_ **(B)**, and EEEV PRNT_50_ **(C)**. On Day 28, the geometric mean MVA-WEEV PRNT_50_ was significantly greater than the Trivalent and TBS control groups and on Day 42, the MVA-WEEV geometric mean PRNT_50_ was significantly greater than the TBS group (p < 0.01). On Day 14, the geometric mean MVA-EEEV PRNT_50_ was significantly greater than the Trivalent group and TBS control (p < 0.0001). On Day 42, the MVA-EEEV geometric mean PRNT_50_ was significantly greater than the TBS control (p < 0.01). Five animals per group were evaluated at each time point for immune responses.

Next, we evaluated the breadth of heterologous protection conferred by the monovalent MVA-VEEV, MVA-WEEV and MVA-EEEV, the Triple-Mix and the Trivalent vaccine against aerosolized alphavirus challenge. For this purpose, female BALB/c mice were vaccinated in the same four-week interval as in the previous study and neutralizing antibodies were measured prior to aerosol challenge with heterologous VEEV (INH-9813), WEEV (Fleming) and EEEV (V105-00210), i.e. EEV strains that differed from the structural proteins encoded in the MVA-based alphavirus vaccines. The majority (9 of 14) of vaccinated animals exhibited a neutralizing antibody response against VEEV four weeks post first vaccination (Study Day 28), and an increased number of seroconverted mice (12 of 14) and increased antibody titers two weeks post second vaccination (**Figure 3A**). The PRNT_50_ range was with 20–1,583 comparable to the first study. In contrast to the first study, the difference in titers elicited by the Triple-Mix compared to mono- or trivalent vaccination was not evident. In fact, there were no statistically significant differences in the VEEV-TrD PRNT_50_ for the different vaccine groups on any study day. Based on the finding that the Triple-Mix vaccine did not reveal any benefit in terms of immunogenicity compared to the Trivalent vaccine, the Triple-Mix was omitted going forward.

Neutralizing antibody responses against WEEV and EEEV were observed as early as two weeks post first vaccination only for the respective monovalent vaccines, i.e. MVA-WEEV or MVA-EEEV (**Figures 3B, C**). By Study Day 42, all five animals in the MVA-WEEV group had seroconverted and three of four animals vaccinated with the Trivalent vaccine exhibited a WEEV PRNT_50_ titer. Similarly, three of four animals in the MVA-EEEV and Trivalent groups exhibited an EEEV PRNT_50_ titer on Study Day 42. On that day, the MVA-WEEV and MVA-EEEV induced geometric mean PRNT_50_ were significantly greater than the TBS control (p < 0.01). While the group mean titers against VEEV and WEEV on that day were also trending higher in the monovalent vaccine groups (162 and 74, respectively) compared to the Trivalent group (44 and 18, respectively), this was not the case for EEEV neutralizing titers with a GMT of 100 (MVA-EEEV) versus 208 (Trivalent).

**Figure 4:**
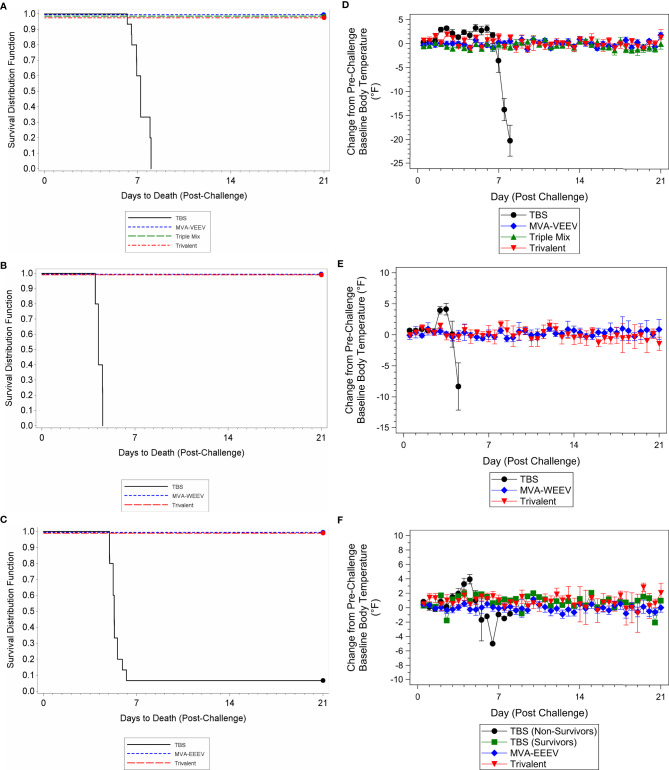
Survival and Body Temperature Changes after VEEV INH-9813, WEEV Fleming, or EEEV V105-00210 Aerosol Challenge of Immunized Mice. Mice were immunized (IM) twice (Day 0 and 28) with the respective vaccine or TBS and then exposed to an aerosolized dose of VEEV, WEEV, or EEEV on Day 42. Kaplan-Meier survival curves of VEEV **(A)**, WEEV **(B)**, and EEEV **(C)** challenge results are shown. There was a significant difference in survival between the TBS group and the vaccination groups (p < 0.01). Group mean changes (with 95% confidence intervals) in body temperature for VEEV **(D)**, WEEV **(E)**, and EEEV **(F)** challenge groups during the post-challenge period are shown. TBS control group mean body temperatures were significantly greater than baseline (p < 0.05) over the first week post-challenge. Ninety-five percent confidence intervals are not included when number of animals was less than three. Ten animals per group were evaluated for survival and body temperature changes.

Although some vaccinated animals did not exhibit a measurable neutralizing antibody response prior to challenge, all animals in the monovalent and Trivalent group (and Triple-Mix against VEEV INH-9813) survived the respective challenge with VEEV INH-9813, WEEV Fleming, and EEEV V105-00210, while 100% of the TBS treated control animals succumbed to VEEV and WEEV, and 90% to EEEV exposure (**Figures 4A–C)**.

The vast majority (greater than 50%) of vaccinated animals were observed as normal throughout the post-challenge period (only rare observations noted that resolved prior to the end of the study) with no changes in body weights (**Supplementary Figures 1B–D**) and body temperature (**Figures 4D–F**) throughout the post-challenge period, while TBS control animals were observed with abnormal clinical observations (e.g. lacrimation, ruffled hair coat, and hunched posture), a group mean decrease in body weight (**Supplementary Figures 1B–D**), and elevated body temperatures following challenge followed by a decrease until the animals succumbed (**Figures 4D–F**), with the exception of the two control animals that survived EEEV challenge. These two survivors initially exhibited elevated body temperatures that resolved compared to the animals that succumbed. Lastly, also consistent with the first study, while several TBS control animals were viremic four days post-exposure (Day 46) or at the terminal time point [viral load ranges 1.64x10^6^ to 2.39x10^7^ pfu/ml (VEEV), 2.00x10^2^ to 2.67x10^2^ pfu/ml (WEEV), and 1.33x10^2^ to 4.14x10^3^ (EEEV)], viremia was not detected for any animals in the respective vaccine candidate groups at any time point during the post-challenge period; thereby providing further support for complete protection conferred by the vaccine candidates.

## Discussion

The primary objective of the studies described here was to evaluate and compare the protective efficacy of different MVA based EEV vaccine approaches against homologous challenge with VEEV (TrD) and heterologous challenge with VEEV (INH-9813), WEEV (Fleming), or EEEV (V105-00210) using stringent inhalation challenge models that are consistent with the anticipated route of infection in an intentional release.

The three vaccine approaches were (1) monovalent alphavirus vaccines MVA-VEEV, MVA-WEEV and MVA-EEEV, (2) a mixture of the three monovalent MVA-based EEV vaccines (Triple-Mix) as first attempt to generate a multivalent vaccine and (3) the true multivalent alphavirus vaccine MVA-WEV (Trivalent) that encodes the polyproteins of all three EEVs in a single vector.

Strikingly, all vaccinated mice survived all alphavirus challenges (VEEV TrD, VEEV INH-9813, WEEV Fleming and EEEV V105-00210) independent of the vaccine approach used. Furthermore, all vaccinated animals in the first study and the vast majority of vaccinated animals in the second study were observed as normal and exhibited no change in body temperature and no body weight loss during the post-challenge period, thereby indicating that the vaccinations prevented morbidity as well. Viremia was not detected in serum assessed at any post-challenge time point for animals vaccinated with the monovalent, Triple-Mix, or Trivalent vaccine, in contrast to TBS control serum.

In addition to the primary objective of assessing protective efficacy, the immunogenicity of the MVA alphavirus vaccines was evaluated and compared, including T cell responses. Overall, there was a wide range in the magnitude of alphavirus-neutralizing antibody responses in all vaccinated groups. Complete (100%) seroconversion after two vaccine administrations was observed for the monovalent vaccines MVA-VEEV and MVA-WEEV in terms of VEEV- and WEEV-specific neutralizing antibodies, respectively, as well as for the Trivalent vaccine in terms of VEEV (TrD)-neutralizing antibodies. However, one mouse of the MVA-EEEV vaccinated group had no detectable EEEV neutralizing antibodies, one mouse each of the Trivalent vaccinated groups did not seroconvert for EEEV or WEEV neutralizing antibodies and 5 of 10 Triple-Mix vaccinated mice in the first study had no measurable VEEV neutralizing antibodies. Irrespective of the detection of neutralizing antibodies, 100% of vaccinated mice were protected against the respective challenge virus. This could be due to the sensitivity of the PRNT assay, protection in the absence of neutralizing antibodies, and/or other factors. The notion that a PRNT assay is not necessarily able to detect all neutralizing antibodies is supported by the total lack of measurable VEEV (TrD)- and EEEV-neutralizing titers we reported earlier (28) after vaccination with Trivalent MVA vaccine, while in the studies performed here, all 10 mice in the Trivalent vaccine group showed VEEV (TrD)-neutralizing titers and 4 of 5 mice were positive in the EEEV specific PRNT assay, indicating that dependent on the assay used, results for the same vaccine may differ.

Protection in the absence of measurable neutralizing antibodies is in line with data from a triple mix alphavirus replicon vaccine, which failed to induce seroconversion in all NHPs, while protecting them against aerosolized EEV challenges (41). Similarly, measurable neutralizing antibodies failed to correlate with protection against EEEV in macaques vaccinated with Sindbis/EEE virus (31) and did not correlate with protection against VEEV-IE in NHP vaccinated with a live attenuated VEEV-IAB virus (30).

Another explanation for protection in the absence of measurable neutralizing antibodies is supported by Bennett (29), Hart (42) and Brooke (37), who report based on mouse studies that cell-mediated immunity in form of cytotoxic T cells may contribute to protection against EEV. In our first study, an IFNɣ T cell response to both the VEEV TrD E1 and E2 peptide pools was observed for all three vaccine groups by Study Day 28, which increased in magnitude on Study Day 42 (after the second vaccination). In fact, 100% of vaccinated mice elicited a robust VEEV-specific IFNɣ T cell response for every vaccine approach used. Thus, our data support the notion that T cells could be involved in protection. However, further studies are necessary to draw firm conclusions about the mechanism of protection. Future studies may also want to assess total alphavirus specific antibody responses by ELISA and/or mucosal antibody responses, since these have also been reported to be involved in protection against EEV (32, 43–45).

Although all MVA based EEV vaccine approaches were equally (100%) effective in terms of protection, not all seemed equally immunogenic in each assay. Our results indicated a significantly lower VEEV-specific mean PRNT_50_ and IFNɣ T cell response specific for the E1 peptide pool induced by the Triple-Mix vaccine compared to the monovalent or Trivalent vaccine in the first study. However, E2 specific T cell responses were comparable in each vaccination group and the significant difference in VEEV-specific mean PRNT_50_ titers could not be reproduced in the second study. It is therefore not completely clear whether the Triple-Mix vaccine approach suffers from immune interference, a finding previously reported for a few other alphavirus vaccine candidates, including a VEEV vaccine that suppressed antibody responses to formalin-inactivated WEEV and EEEV vaccines in humans and equines (34, 35). Lower immune responses were not observed when simultaneous expression of the three EEV antigens within one vector was used, since mice vaccinated with the Trivalent vaccine elicited a similar magnitude of VEEV-specific neutralizing antibodies after the second vaccination compared to the monovalent vaccine. While the overall trend was slightly lower compared to MVA-WEEV, the antibody responses to EEEV induced by the Trivalent vaccine were again similar compared to those afforded by monovalent MVA-EEEV.

Taken together, all three MVA based vaccine approaches promoted both a humoral and cellular response and were highly efficacious against otherwise lethal infections with aerosolized EEV in mice. This was true even with challenge viruses VEEV INH-9813, WEEV Fleming, or EEEV V105-00210, whose envelope polyproteins differ by 13, 19 and 1 amino acid(s) from the E3-E2-6k-E1 polyproteins VEEV TrD, WEEV 71V-1658, and EEEV FL93-939NA, respectively, encoded by the vaccines. Mixing MVA-VEEV, MVA-WEEV, and MVA-EEEV (Triple-Mix) appeared to be a first feasible approach for a vaccine that protects against all three EEVs. However, the benefit of MVA to carry a high load of foreign genes was realized in the Trivalent vaccine MVA-WEV that encodes the polyprotein of all three EEVs in a single MVA vector and that was completely protective without any signs of immune interference. Together with the excellent safety profile of the MVA vector that is licensed in Europe, Canada and the US as smallpox vaccine, MVA-WEV (Trivalent) could overcome safety and efficiency issues encountered with attenuated and inactivated EEV vaccines, respectively, and thereby represents a promising vaccine candidate able to protect against all three equine encephalitis viruses.

## Data Availability Statement

The original contributions presented in the study are included in the article/**Supplementary Material**; further inquiries can be directed to the corresponding author.

## Ethics Statement

The animal study was reviewed and approved by IACUC and Animal Care and Use Review Office (ACURO).

## Author Contributions

KE, RS, and AV contributed to the conception and design of the study. LH wrote the first draft of the manuscript. KE and AV contributed to the manuscript revision. MA performed the statistical analysis. All authors contributed to the article and approved the submitted version.

## Funding

The work was funded under the JPM CBRN Medical contract MCDC-17-04-001 and GS00Q140ADU402.

## Conflict of Interest

KE, RS, and AV are employees of Bavarian Nordic GmbH, Germany. RS is an inventor on pending patent applications of Bavarian Nordic A/S, Denmark.

The remaining authors declare that the research was conducted in the absence of any commercial or financial relationships that could be construed as a potential conflict of interest.

## Supplementary Material

Supplementary Figure 1 Body Weight Changes after VEEV TrD, VEEV INH-9813, WEEV Fleming, or EEEV V105-00210 Aerosol Challenge of Immunized Mice. Ten mice per group were immunized (IM) twice (Day 0 and 28) with the respective monovalent vaccine, Triple-Mix, Trivalent or TBS as indicated and then exposed to an aerosolized dose of VEEV TrD **(A)**, VEEV INH-9813 **(B)**, WEEV **(C)**, or EEEV **(D)** on Day 42.
