# Supplementary figures and images for: A Monovalent and Trivalent MVA-Based Vaccine Completely Protects Mice Against Lethal Venezuelan, Western, and Eastern Equine Encephalitis Virus Aerosol Challenge
Source: Front Immunol. 2021 Jan 19;11:598847. doi: 10.3389/fimmu.2020.598847 (PMC7851092; doi:10.3389/fimmu.2020.598847)

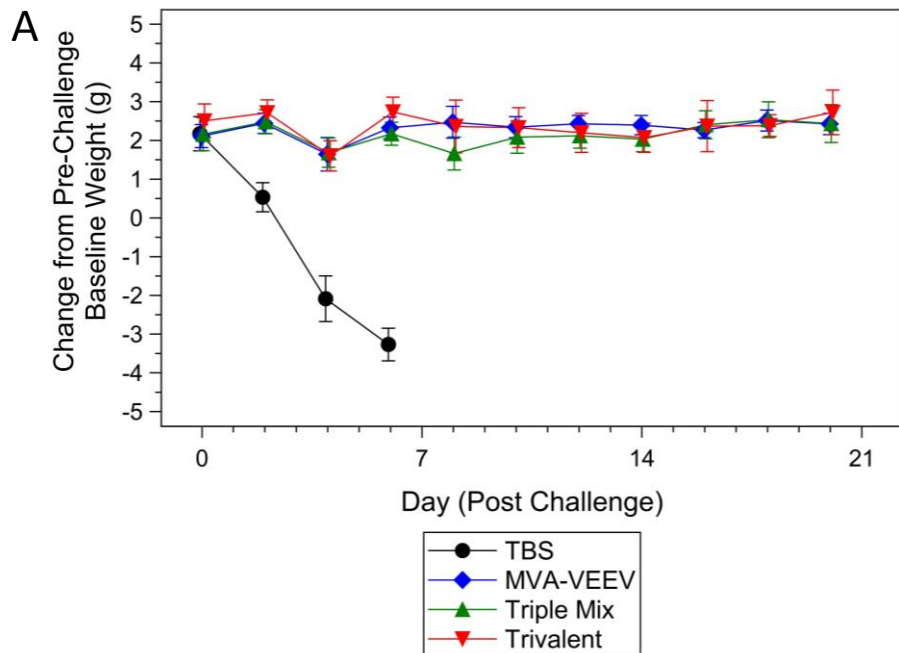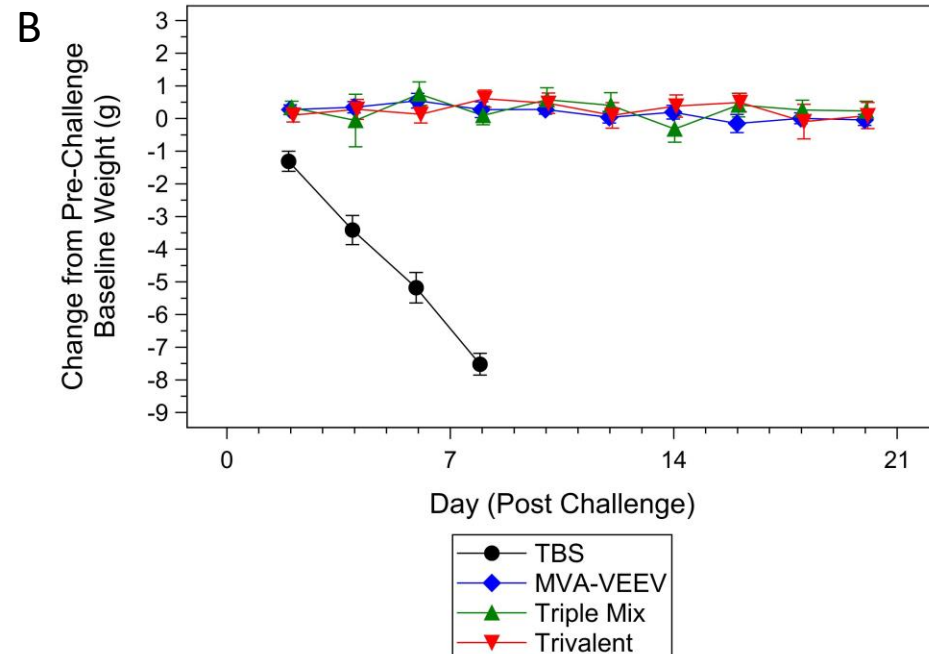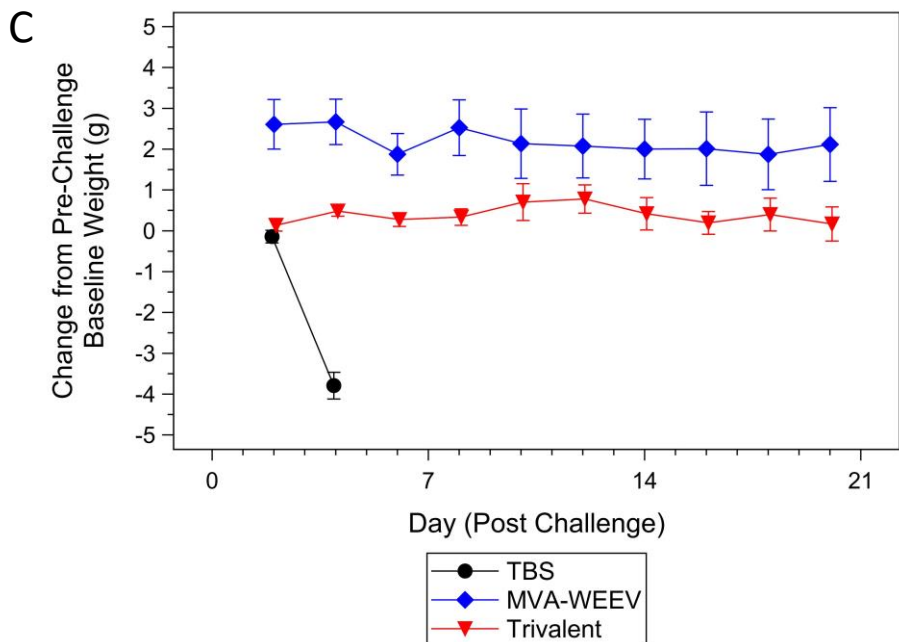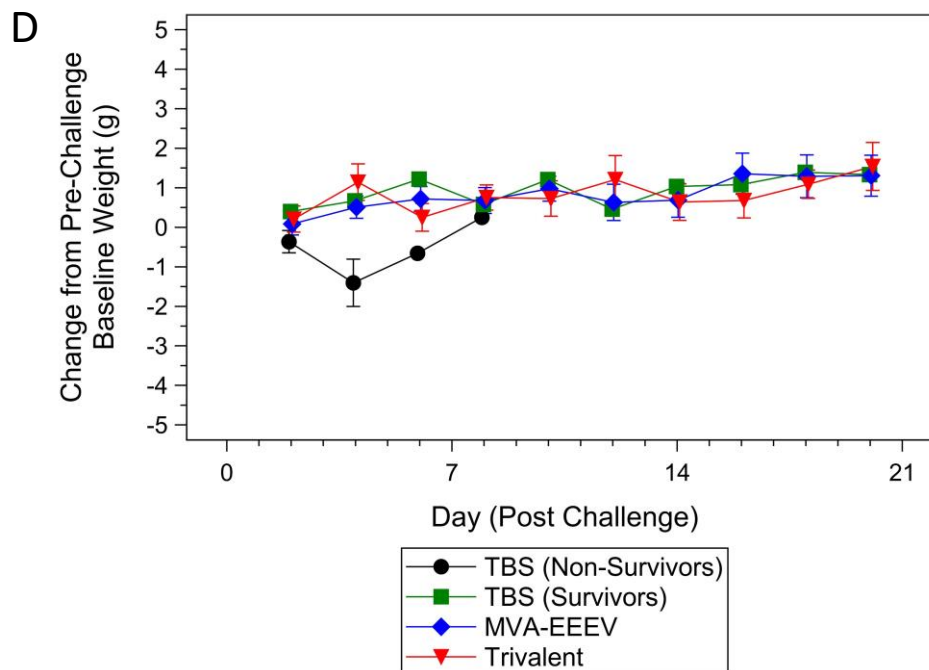

Supplement: Supplementary Figure 1 — Body Weight Changes after VEEV TrD, VEEV INH-9813, WEEV Fleming, or EEEV V105-00210 Aerosol Challenge of Immunized Mice. Ten mice per group were immunized (IM) twice (Day 0 and 28) with the respective monovalent vaccine, Triple-Mix, Trivalent or TBS as indicated and then exposed to an aerosolized dose of VEEV TrD (A), VEEV INH-9813 (B), WEEV (C), or EEEV (D) on Day 42. [file Image_1.pdf]
